# A requirement for hedgehog signaling in thyroid hormone-induced postembryonic intestinal remodeling

**DOI:** 10.1186/s13578-015-0004-3

**Published:** 2015-03-24

**Authors:** Luan Wen, Takashi Hasebe, Thomas C Miller, Atsuko Ishizuya-Oka, Yun-Bo Shi

**Affiliations:** Section on Molecular Morphogenesis, Program on Cell Regulation and Metabolism, National Institute of Child Health and Human Development, National Institutes of Health, Bldg. 18 T, Rm. 106, Bethesda, MD 20892 USA; Department of Biology, Nippon Medical School, 1-7-1 Kyonan-cho, Musashino, Tokyo 180-0023 Japan

**Keywords:** *Xenopus laevis*, Thyroid hormone receptor, Amphibian metamorphosis, Adult stem cells, Postembryonic development

## Abstract

**Background:**

Intestinal remodeling during amphibian metamorphosis has long been studied as a model for the formation of the adult organs in vertebrates, especially the formation of adult organ-specific stem cells. Like all other processes during metamorphosis, this process is controlled by thyroid hormone (T3), which affects cell fate and behavior through transcriptional regulation of target genes by binding to T3 receptors (TRs). Earlier studies have shown that Sonic hedgehog (Shh) is induced by T3 in the developing adult stem cells and that the Shh receptor and other downstream components are present in the connective tissue and at lower levels in the muscles at the climax of intestinal remodeling. However, no *in vivo* studies have carried out to investigate whether Shh produced in the adult cells can regulate the connective tissue to promote intestinal maturation.

**Results:**

We have addressed this issue by treating tadpoles with Shh inhibitor cyclopamine. We showed that cyclopamine but not the structurally related chemical tomatidine inhibited the expression of Shh response genes BMP4, Snai2, and Twist1. More importantly, we showed that cyclopamine reduced the cell proliferation of both the developing adult stem cells as well as cells in the other intestinal tissues at the climax of metamorphosis, leading to delayed/incomplete remodeling of the intestine at the end of metamorphosis. We further revealed that both Snai2 and Twist1 were strongly upregulated during metamorphosis in the intestine and their expression was restricted to the connective tissue.

**Conclusions:**

Our results suggest that Shh indeed signals the connective tissue whereby it can increase adult stem cell proliferation and promote formation of the adult intestine.

## Background

Thyroid hormone (T3) plays a critical role during postembryonic development in vertebrates. In mammals, this refers to the period around birth when T3 levels peak in the plasma and when many organs mature into the adult form [1-4]. Due to the difficulty to manipulate mammalian embryos and separate maternal effects from endogenous regulation it has been difficult to study mechanisms underlying the maturation of the adult organs, including the development of organ-specific adult stem cells and how T3 affects this process [2,3,5]. Amphibian metamorphosis resembles mammalian postembryonic development because of both the high levels of T3 in the plasma and similar organ maturation/remodeling processes during this period [2,3,5]. Metamorphosis, however, offers a unique advantage of being able to be easily manipulated by adding T3 inhibitors or physiological levels of exogenous T3 in the tadpole rearing water. This makes it an excellent model to study both the *in vivo* mechanisms of T3 action and the formation of the adult organs, particularly adult organ-specific stem cells [2,3,6-10].

During metamorphosis, essentially every organ/tissue undergoes extensive changes [3]. The tadpole intestine remodels drastically, transforming from a simple tubular organ of mostly larval epithelial cells with little connective tissue or muscles, to a complex organ with a multiply folded adult epithelium supported by thick layers of connective tissue and muscles [11]. This involves almost complete removal of larval epithelial cells through apoptosis and *de novo* formation of adult stem cells, which express well-established markers of adult intestinal stem cells in mammals [9,11-13]. Earlier studies have shown that the adult epithelial stem cells originate through dedifferentiation of some larval epithelial cells in a process that requires T3 action in both the epithelium and the surrounding non-epithelium, most likely the connective tissue [9,14-17].

T3 affects target gene transcription by binding to T3 receptors (TRs). TRs are members of the nuclear hormone receptor superfamily, which also includes 9-cis retinoic acid receptors (RXRs). For T3 inducible genes, TRs function as heterodimers with RXRs to bind to T3 response elements (TREs) in T3-target genes constitutively, and repress or activate their transcription in the absence or presence of T3, respectively [1,8,18-30]. These direct target genes in turn affect the expression of downstream T3 response genes. Numerous T3 target genes in the intestine of *Xenopus laevis*, the most widely studied amphibian species, have been identified by using various methods [31-35]. Among them is Sonic hedgehog (Shh), which is directly regulated by T3 at the transcription level since its upregulation by T3 in premetamorphic tadpoles does not require new protein synthesis [36,37].

Shh is the best-studied member of the three hedgehog genes encoding secreted proteins that affect cell-fate and behavior in diverse tissues and processes [38-40]. Shh binds to the 12-transmembrane receptor Patched (Ptc) [41]. This binding relieves Ptc-mediated inhibition of Smoothened (Smo), another transmembrane protein, leading to the activation of Shh target genes via transcription factors Gli1, 2, 3 downstream in the Shh signaling pathway [38-40,42,43]. We have previously shown that both Shh and the downstream factors of the pathway are all upregulated in the intestine during *Xenopus laevis* metamorphosis. Shh is expressed in the developing adult epithelial stem cells while the downstream factors are expressed predominantly in the connective tissue with weak levels in the muscles [44]. Importantly, organ culture studies of premetamorphic intestine have shown that Shh stimulates the proliferation of cells in both the epithelial and non-epithelial tissues in the absence of T3. These suggest that Shh acts by signaling the non-epithelial tissues to affect intestinal remodeling.

Here, we have investigated the effect of endogenous Shh signaling on the intestine during metamorphosis by using Shh inhibitor cyclopamine. We showed that Shh signaling is required for the formation and/or proliferation of adult epithelial stem cells as well as the upregulation of Shh response genes in the connective tissue. We have further revealed that the expression of the Shh response genes Snai2 and Twist1 in the connective tissue is spatiotemporally correlated with the development of the adult epithelium. Thus, our results suggest that Shh signals the connective tissue, which in turn facilitates the development of the adult intestinal epithelium.

## Results

### Inhibition of hedgehog (Shh) signaling by cyclopamine suppresses intestinal remodeling during *Xenopus laevis* metamorphosis

**Figure 1:**
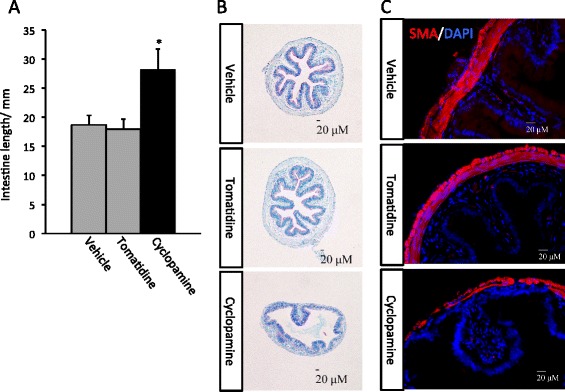
**Inhibition of hedgehog (Shh) signaling by cyclopamine suppresses intestinal remodeling during** ***Xenopus laevis*** **metamorphosis** ***.*** **A**: Cyclopamine-treated animals have longer intestine at the end of metamorphosis. Tadpoles at stage 58 were treated with vehicle (100% ethanol, final concentration in rearing water: 0.1%) (3 tadpoles) or tomatidine (7 tadpoles), or cyclopamine (8 tadpoles) till they reached stage 66. Intestinal length was measured from bile duct junction to colon for all animals. * indicated significant difference between the cyclopamine group and the other two groups (p < 0.05). Note that the cyclopamine-treated tadpoles completed metamorphosis, as judged based on external morphology, e.g., resorption of the tail, similarly as the animals in the tomatidine and vehicle groups. However, they had significant longer intestine. **B**: Retarded intestinal maturation in the cyclopamine-treated animals. Transverse sections of the intestine from the animals above were stained with methyl green pyronin Y (MGPY), which stained DNA in blue (methyl green) and RNA in red (pyronin Y). Note that the cyclopamine-treated animals had intestines with fewer and less structured epithelial folds as well as reduced connective tissue and muscles (outer layers). **C**: Immunofluorescence staining showing reduced intestinal muscle development in the cyclopamine-treated animals. The intestinal sections above were stained with anti-smooth muscle actin (SMA) antibody as well as DAPI for cell nuclei.

To investigate the role of endogenous Shh signaling during metamorphosis, we treated tadpoles at stage 58, early climax of metamorphosis when upregulation of endogenous Shh begins [36,45], with two structurally related chemicals. One of them, cyclopamine, specifically inhibits Shh signaling by binding to Smo [46], while the second one, tomatidine, has no effect on Shh signaling [47]. When vehicle-treated control tadpoles reached the end of metamorphosis (stage 66, about 1 week after the start of the treatment at room temperature), the animals were sacrificed for analysis. Morphologically, the control and the drug treated groups reached the end of metamorphosis similarly (data not shown), suggesting that neither drug has adverse effects on T3 action or gross development of the animals. On the other hand, the total length of the intestine from the tadpoles treated with cyclopamine was significantly longer than that from the control and tomatidine-treated groups (Figure 1A). The intestine is known to undergo drastic reduction in length, with as much as 90% reduction for the small intestine, during metamorphosis [48]. The longer intestine in the cyclopamine-treated group suggested that cyclopamine treatment delayed the remodeling of the intestine into the adult form, even though most other organs appeared to metamorphosed normally based on external morphology of the animals. To investigate this further, we sectioned the intestine and stained the sections with methyl green-pyronin Y (MGPY), which stained DNA in blue (methyl green) and RNA in red (pyronin Y) [49]. As shown in Figure 1B, the control and tomatidine-treated groups had a typical adult intestinal morphology, with a multiply folded epithelium surrounded by thick layers of connective tissue and muscles. In contrast, inhibiting Shh signaling with cyclopamine resulted in a less developed intestine, with fewer and smaller intestinal epithelial folds resembling the intestine of wild type tadpoles around stage 63 [48]. Furthermore, immuno-staining with anti-smooth muscle actin (SMA) antibody demonstrated clearly that cyclopamine-treated animals also had reduced intestinal muscles compared to the other two groups (Figure 1C). With all different analyses, we found that tomatidine-treated group behaved identically as the control, vehicle-treated group, suggesting that the effects of cyclopamine was due to its inhibition of Shh signaling. Thus, Shh signaling is required for the remodeling of all three major tissues in the intestine during metamorphosis.

**Figure 2:**
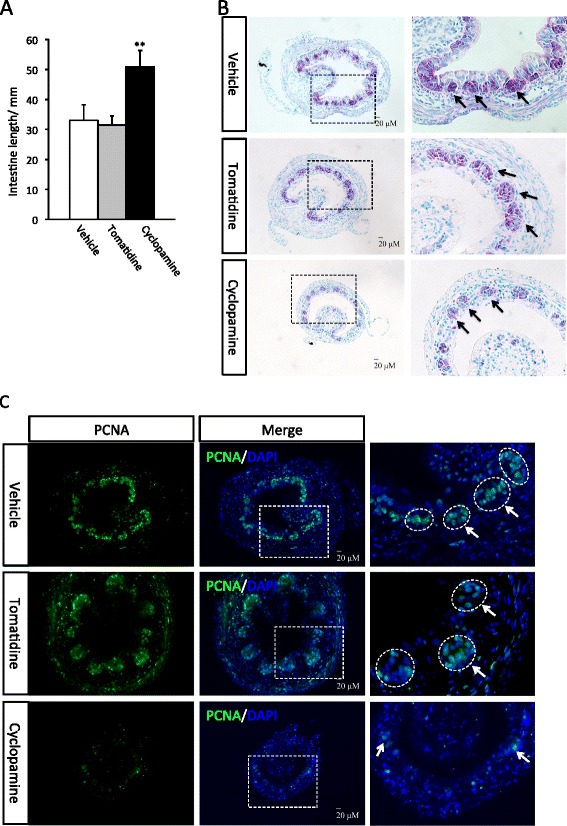
**Inhibition of hedgehog signaling suppresses intestine shortening and remodeling at metamorphic climax. A**: Cyclopamine-treated tadpoles have longer intestine at the climax. Tadpoles at stage 58 were treated with vehicle (100% ethanol, final concentration in rearing water: 0.1%) (9 tadpoles) or tomatidine (10 tadpoles), or cyclopamine (11 tadpoles) till they reached climax (stage 62). Intestine was isolated and measured from bile duct junction to colon. The animals in all three groups reached developed to stage 62 similarly as judged based on external morphology. ** indicate significant difference between the cyclopamine group and the two control groups (p < 0.001). **B**: Cyclopamine-treated tadpoles have fewer islets of adult epithelial stem cells. Transverse sections of the intestine from above were stained with methyl green pyronin Y (MGPY). The boxed area in the left panel is enlarged in the right panel. Note that at the climax of metamorphosis, the clusters (islets) of adult epithelial stem cells are stained strongly by MGPY (arrows) while the dying larval epithelial cells are only weakly stained [15,49,50]. The cyclopamine-treated tadpoles had smaller intestinal cross-section and fewer islets of adult cells. **C**: Cyclopamine-treated tadpoles have reduced cell proliferation. The intestinal sections above were stained with DAPI for nuclear DNA and anti-PCNA antibody for mitotic cells. The boxed area in the middle panel is enlarged in the right panel. Cyclopamine treatment significantly reduced cell proliferation in the intestine, especially in the epithelium. Note that the labeling in the epithelium was limited to the islet of the adult cells (arrows) but not in the dying larval epithelial cells, as expected.

To investigate how cyclopamine affects the remodeling of the intestine, we treated tadpoles at stage 58 as above and analyzed them when they reached the climax (stage 61/62). Again, the animals in all three groups reached the climax similarly (not shown) and the cyclopamine-treated tadpoles had longer intestine than the other two groups (Figure 2A). We next stained the intestinal sections with methyl green-pyronin Y (MGPY). It has been well established that at the climax of metamorphosis (stage 61/62), clusters of adult epithelial stem cells (islets) are stained strongly red with MGPY, while the dying larval epithelial cells are only weakly stained [15,49,50]. Consistently, we observed that as expected, the control and tomatidine-treated tadpole intestine had numerous islets of adult epithelial stem cells (Figure 2B). In contrast, the cyclopamine-treated tadpoles had smaller intestinal cross-sections and fewer islets of adult stem cells (Figure 2B). When the intestinal sections were stained with anti-PCNA antibody for mitotic cells, we observed that cyclopamine treatment significantly reduced cell proliferation in the intestine, especially in the epithelium (Figure 2C). Thus, Shh signaling is important for cell proliferation during intestinal remodeling.

### The expression of Shh target genes is blocked by cyclopamine

**Figure 3:**
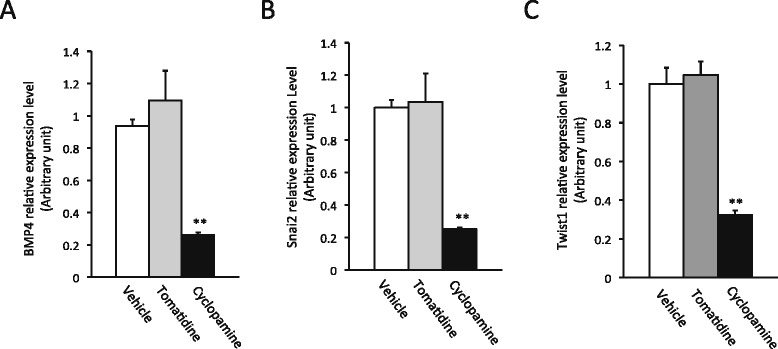
**Cyclopamine inhibits Shh target gene expression.** Tadpoles at stage 58 were treated as in Figure 2 until they reached stage 62. Total intestinal RNA was isolated for qRT-PCR analysis of the expression of Shh target genes BMP4 [51] **(A)**, Snai2 [52] **(B)** and Twist1 [52] **(C)**. ** indicate significant difference between the cyclopamine group and the other groups (p < 0.001).

Cyclopamine is known to be a specific inhibitor of Shh signaling. To determine if this was indeed the mechanism of its action in the intestine during metamorphosis, we analyzed the expression of several well known Shh target genes: BMP4 [51], Snai2 and Twist1 [52]. Of the three, BMP4 and Snai2 are known to be upregulated at the climax of intestinal metamorphosis when Shh expression peaks [33,51], suggesting that they are regulated by Shh during metamorphosis. Indeed, when we treated tadpoles at stage 58 till they reached climax (stage 61/62) and isolated intestinal RNA for gene expression analysis, we observed that the expression of all three target genes were inhibited by cyclopamine but not tomatidine (Figure 3), indicating that cyclopamine specifically inhibits Shh signaling.

### The Shh target genes Snai2 and Twist1 are expressed exclusively in the connective tissue and their mRNA levels are upregulated during intestinal metamorphosis

**Figure 4:**
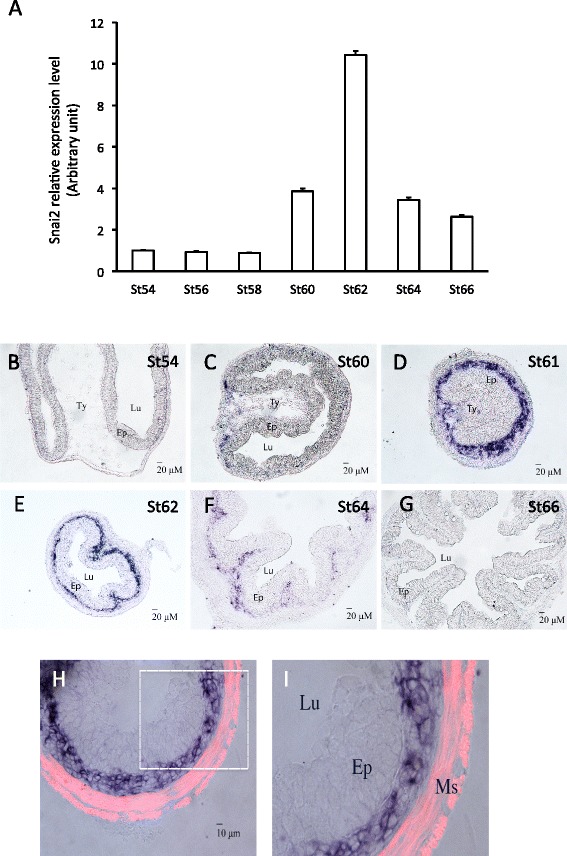
**Snai2 expression is restricted to the connective tissue and peaks at the climax of intestinal metamorphosis. A**: Snai2 mRNA level peaks at the climax (Stage 62). Quantitative RT-PCR analysis of Snai2 mRNA level in intestine from tadpoles at indicated stages, normalized against the control gene EF1a. **B**-**G**: In situ hybridization reveals connective tissue-specific expression of Snai2 in intestine. Note that high levels of Snai2 mRNA were detected fairly uniformly in the connective tissue at the climax (St61-62). **H**-**I**: Snai2 is not expressed in the muscle layers. In situ hybridization sections of Snai2 at the stage 61 were double-stained with smooth muscle actin antibody (red) to label the muscle layers (Ms). Ty: typhlosole, the single epithelial fold in in premetamorphic tadpole intestine where connective tissue is abundant; Lu: lumen; Ep: epithelium. Note that Snai2 labeled cells are localized between epithelium and muscle layers.

**Figure 5:**
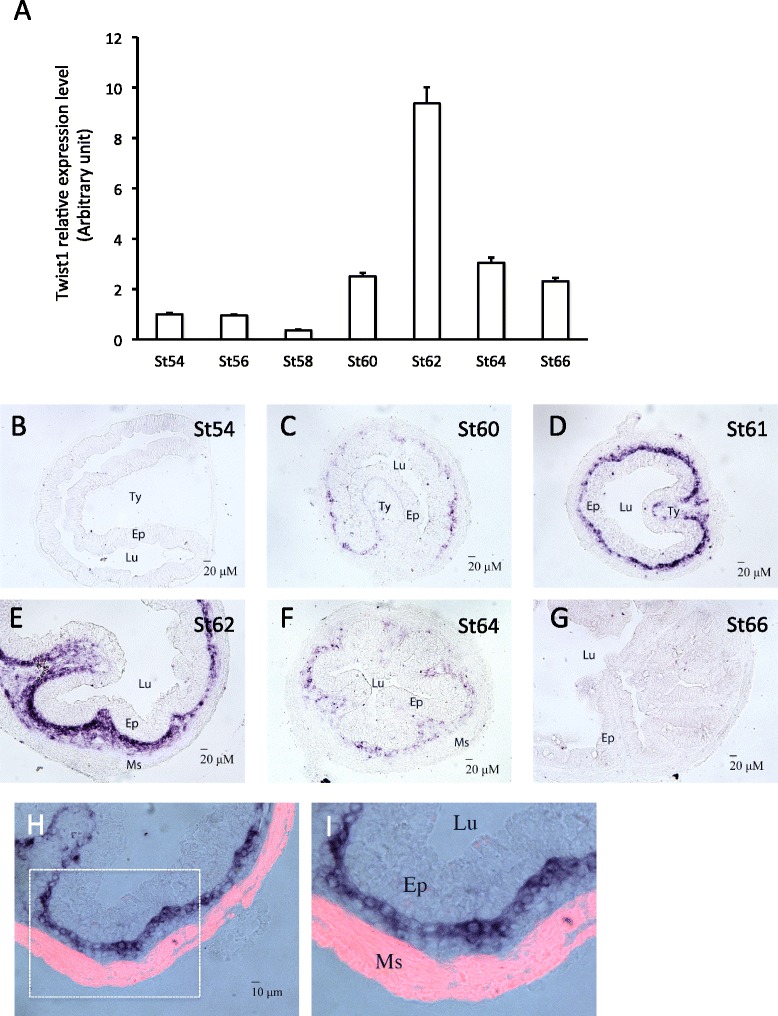
**Twist1 has similar spatiotemporal expression profiles as Snai2 in the intestine. A**: Twist1 mRNA level peaks at the climax (Stage 62). Quantitative RT-PCR analysis of Twist1 mRNA level in intestine from tadpoles at indicated stages, normalized against the control gene EF1a. **B**-**G**: In situ hybridization reveals connective tissue-specific expression of Twist1 in intestine. Note that Twist1 mRNA level peaked in the connective tissue at the climax (St61-62). **H**-**I**: Twist1 is not expressed in the muscle layers. In situ hybridization sections of Twist1 at the stage 61 were double-stained with smooth muscle actin antibody (red) to labeled the muscle layers (Ms). Ty: typhlosole, the single epithelial fold in in premetamorphic tadpole intestine where connective tissue is abundant; Lu: lumen; Ep: epithelium. Note that Twist1 labeled cells are localized between epithelium and muscle layers.

Shh is a direct T3 target gene and its expression is limited to the epithelium during intestinal remodeling [36,45]. On the other hand, the downstream receptors and transcription factors of the Shh signaling pathway are upregulated and strongly expressed mainly in the connective tissue with some weak expression in the muscles [44], suggesting that Shh functions mainly by inducing target genes in the connective tissue. To test this possibility, we next determined the spatiotemporal expression profiles of Snai2 and Twist1, we first isolated total RNA from the intestine of tadpoles at different stages of metamorphosis, from premetamorphic stage 54 to the end of metamorphosis (stage 66) and analyzed the expression of Snai2 and Twist1 by RT-PCR. The results showed that the expression of both genes paralleled that of Shh [36]. That is, both had little expression prior to metamorphosis and their mRNA levels were upregulated by stage 60 and peaked around stage 62 (Figures 4A and 5A).

In situ hybridization showed that both genes were expressed in the connective tissue but not the epithelium at the climax of metamorphosis with little mRNA in premetamorphic (stage 54) or postmetamorphic (stage 66) intestine (Figure 4B-G and Figure 5B-G). Double-staining with smooth muscle actin antibody demonstrated that the expression was also absent in the muscles (Figures 4H,I and 5H,I), indicating that Snai2 and Twist1 were expressed exclusively in the connective tissue at the climax of intestinal metamorphosis.

## Discussion/conclusion

Intestinal remodeling during frog metamorphosis offers an excellent opportunity to study the formation of adult organ-specific stem cells during vertebrate development [53,54]. Studies on intestinal remodeling have led to the identification of many candidate genes likely important for the formation of adult stem cells. Furthermore, comparative analysis of the TR coactivator PRMT1 (protein arginine methyltransferase 1) in frog, mouse and zebrafish has suggested that the formation of adult intestinal stem cells is evolutionally conserved and takes place during the postembryonic period when T3 levels are high [50]. Subsequent genetic analyses in mouse provided further support that adult intestinal stem cells are distinct from embryonic ones and are formed during the postnatal period in mouse when T3 levels rise [55,56]. We have previously demonstrated that cell-cell interactions induced by T3 are essential for the formation of adult intestinal stem cells during metamorphosis [14]. Our studies here suggest that Shh pathway functions to mediate the T3-induced cell-cell interactions important for the formation of the adult intestine during metamorphosis.

Shh was first discovered as a direct T3 target gene in the intestine during *Xenopus laevis* metamorphosis [36,37]. Subsequently, it was shown that Shh was specifically upregulated in the developing adult epithelial stem cells [45], suggesting a role of Shh in intestinal remodeling. Interestingly, the addition of Shh to organ cultures of premetamorphic intestine leads to increased cell proliferation in both the larval epithelium and non-epithelial tissues even in the absence of T3 [45]. In the presence of T3 the effects of exogenous Shh were minimal, likely because endogenous Shh induced by T3 was sufficient. In the absence of T3, all cells in the organ cultures of the larval intestine are likely of the larval types. In addition, there are no larval epithelial stem cells in premetamorphic tadpole intestine and the differentiated larval epithelial cells are mitotically active to serve the function of self-renewal. These findings suggest that Shh is able to stimulate the proliferation of even the larval cells in different intestinal tissues in the tadpoles, at least in organ cultures.

As a secreted protein, Shh can signal both the epithelium as well as other tissues. Interestingly, all other members of the Shh signaling pathway, including Shh receptor Patched and downstream transcription factors, are all strongly upregulated in the connective tissue at the climax of intestinal metamorphosis when stem cell formation and proliferation take place [44]. Only weak expression for these genes is present in the muscles. In addition, one of the known Shh target genes, BMP4, was found to be strongly induced during metamorphosis in the connective tissue of the intestine [51]. Our studies here showed that two other Shh targets, Snai2 and Twist1, likewise, also expressed exclusively in the connective tissue at the climax of metamorphosis. These results suggest that exogenous Shh enhances cell proliferation in the intestinal organ cultures mainly by signaling the connective tissue. However, the role of endogenous Shh signaling during metamorphosis remains unclear.

By treating tadpoles with cyclopamine, we showed here that Shh signaling is required for the development of the adult intestine. Several lines of evidence suggest that cyclopamine specifically inhibited Shh signaling during metamorphosis. First, cyclopamine inhibited the expression of known Shh target genes. Second, Shh induction by T3 during metamorphosis is known to be organ-specific, with strong induction in intestine, weak induction in the head and hindlimb, and no upregulation in the tail [36]. Consistently, we found that cyclopamine treatment had little effect on overall external morphological changes during metamorphosis but had a strong effect on the intestinal remodeling. Third, cyclopamine specifically affected the processes of intestinal remodeling when Shh is highly expressed. Finally, the structurally related compound tomatidine had no effect on either the morphological changes during intestinal remodeling or the expression of the Shh target genes. Thus, our findings suggest that Shh signaling plays an important role for the formation of the adult intestine during metamorphosis.

**Figure 6:**
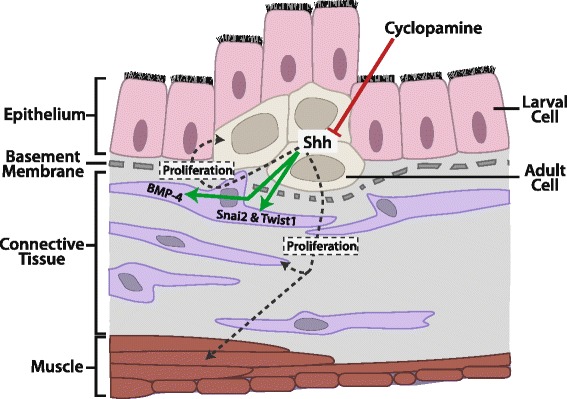
**A schematic diagram showing the effects of Shh signaling during intestinal remodeling.** Shh appears to enhance the proliferation of cells in all three tissue layers during metamorphosis. Shh receptors and downstream transcription factors are highly expressed in the connective tissue, which are likely responsible for the activation of the expression of BMP-4, Snai2, and Twist1. The connective tissue may in turn help to mediate the cell proliferation effects of Shh on the adult epithelial stem cells and the circular muscle cells. Cyclopamine directly inhibits Shh signaling in the epithelium and thus blocks all the effects of Shh.

Complementary to the findings in organ cultures, we observed that blocking Shh signaling in metamorphosing tadpoles reduced cell proliferation in the intestine, particularly the adult epithelial stem cells. These results suggest that Shh signaling is a mediator of T3-idnuced cell-cell interactions important for adult stem cell development. That is, during metamorphosis, T3 induces the expression of Shh in the epithelium and the downstream members of the pathway in the connective tissue (and weakly in the muscles). Shh then binds to the receptor to activate its target genes such as BMP4, Snai2, and Twist1, in the connective tissue (possibly weakly in the muscles, although such target genes are yet to be discovered) (Figure 6). The activation of these target genes likely in turn enhances the proliferation of the cells in the connective tissue (and muscles) as well as signaling back to the developing adult epithelium to increase the proliferation of the adult stem cells through yet unknown mechanism.

Interestingly, blocking Shh signaling also delayed the shortening of the intestine during metamorphosis, suggesting that larval epithelial cell apoptosis was also being affected. It is well known that the small intestine reduces its length by as much as 90% during metamorphosis due to larval epithelial cell death and muscle contraction [11,57]. In addition, cell-cell interaction is known to be able to influencing larval cell death, that is thyroid hormonal signaling in the non-epithelial tissues (connective tissue and muscles) can lead to larval cell death [14,16,58,59]. Thus, it is very likely that Shh inhibition affects connective tissue and/or muscles, which in turn inhibits larval cell death, therefore, leading to increased intestinal length.

With the advent of gene knockdown/knockout technologies for Xenopus [60-63], it should be possible in the future to investigate how the endogenous Shh signaling pathway participates in regulating cell-cell interactions important for both larval cell death and adult stem cell development and/or proliferation. In addition, given the conservation among vertebrate development [53,54], it is very likely that Shh signaling plays a similar role in the formation/maturation of the adult intestine in mammals. While no studies have been done on the role of Shh signaling during postembryonic development in mammals, transgenic inhibition of Shh signaling in the intestine during mouse embryogenesis leads to severe defects in the villus/crypt structure around neonatal stages, indicating a critical role of Shh signaling for intestinal embryogenesis. It would be interesting to determine whether there is a similar requirement for Shh for the formation and proliferation of the adult intestinal stem cells in mammals.

## Methods

### Animal treatments

All animal care and treatments were done as approved by Animal Use and Care Committee of Eunice Kennedy Shriver National Institute of Child Health and Human Development (NICHD), National Institutes of Health (NIH). Adult *Xenopus laevis* were purchased from NASCO. Tadpoles were obtained by breeding wild type frogs, and were staged according to [64].

Stage 58 *Xenopus laevis* tadpoles of similar sizes were treated with cyclopamine (Enzo life sciences) (2.5 μM), tomatidine (Enzo life sciences) (2.5 μM), or vehicle (100% ethanol, final concentration in rearing water: 0.1%), respectively, at room temperature. The rearing water was changed every two days.

### In situ hybridization and immunohistochemistry

Full-length clones of Snai2 and Twist1 were purchased from Open Biosystem. The plasmids pCMV-SPORT-snai2 and pCS111-twist1 were linearized with KpnI and HindIII, respectively. Both Snai2 and Twist1 Digoxigenin labeled anti-sense probes were generated by transcribing the linearized plasmids with T7 RNA polymerase with the DIG RNA Labeling Kit (Roche).

The intestine was isolated from tadpoles at different stages, flushed to remove food residues, and fixed in 4% MEMFA for 2 hours at room temperature. The fixed intestine was incubated in 10% sucrose, 20% sucrose, 30% sucrose and OCT, respectively, for 10 min each, then cut into appropriate length and embedded in OCT for frozen sections. Intestine tissues were sectioned at 10 μm with a cryotome, and dried on slide for 2 hours at 42°C. In situ hybridization was carried out according to previous description [12].

For double staining, sections were fixed in 4% MEMFA for 20 min at room temperature after stopping the in situ hybridization staining. They were then washed in TE buffer (10 mM Tris–HCl, 1mMEDTA, pH8.0) for 5 min twice and treated with 10 μg/ml proteinase K (Roche) in 50 mM Tris–HCl, 5 mM EDTA pH8.0 for 5 min at 37°C. After washing for 5 min in 1 × TBST (Tris buffered saline add 0.1% tween-20) three times, the sections were incubated with blocking buffer (10% normal goat serum in 1 × TBST) at room temperature for 1 hour and immunostained with anti-smooth muscle actin antibody (diluted 1:400; Sigma) at 4°C overnight. They were then washed for 10 min in 1 × TBST three times and incubated with TRITC-labeled secondary antibody (Millipore) for 1 hour at room temperature. The sections were next mounted with DAPI-containing mount medium (Vector). Bright view and fluorescent photographs were taken by using a digital CCD color camera (Retiga Exi, QImaging) attached to an optical microscope (BX60, Olympus).

For methyl green-pyronin Y staining, the fixed intestinal tissues were dehydrated with a tissue processor (Thermo electronic), embedded in paraffin, and were sectioned with a microtome (MICROM HM315, Thermo scientific) at 5 μm. Tissue sections were mounted on poly-lysine coated slides (Fisher Scientific) and were deparaffinized in xylene. After rehydrating in a series of different concentrations of ethanol, they were stained in methyl green-pyronin Y solution (Muto, Tokyo, Japan) for 5 min at room temperature, dehydrated, and were mounted in toluene solution (Thermo Scientific).

Immunofluorescence on the paraffin embedded sections was carried out as previously described [65]. The sections were deparaffinized in xylene, rehydrated in a series of different concentrations of ethanol, and were boiled in the antigen retrieve buffer (1 mM Tris, 1 mM EDTA and 0.05% Tween-20) for 3 min. The sections were then washed in 1 × TBST (Tris buffered saline plus 0.1% tween-20), incubated in the blocking buffer (10% normal goat serum) for 1 hour at room temperature, and treated with the following primary antibodies at 4°C overnight: anti-smooth muscle actin antibody (diluted 1:400; Sigma) and anti-proliferating cell nuclear antigen (PCNA) antibody (1:100; Novocastra). After washing in 1 × TBST several times, the sections were incubated with fluorescent-labeled secondary antibody (Millipore) for 1 hour at room temperature and were mounted with DAPI-containing mount medium (Vector). The fluorescence pictures for different colors and/different sections were taken under the same settings.

### RNA isolation and quantitative PCR

The intestines from three tadpoles were pooled together for RNA isolation with Trizole (Invitrogen). Reverse transcription (RT) was carried out by using a reverse transcription kit (Applied Biosystem). Quantitative RT-PCR were carried out by using the SYBR green method with the forward primer 5′- CTTCTCCGTGTGGAGGATGG -3′ and reverse primer 5′- GGGGATCCAGCTCCAGTTTC -3′ for Twist1 (NM_001085883), forward primer 5′- GCTACACAGCAACCAGATTCCTC -3′ and reverse primer 5′- CGCTAAAGATGCACATCAGGACAC -3′ for Snai2 (NM_001086282), forward primer 5′- AGCCCAGTAAGGATGTGGTG -3′ and reverse primer 5′- GCTGCTGAGGTTGAACACAA -3′ for BMP4 (NM_001088032). The expression of the genes was normalized against the expression of EF1α (elongation factor 1α) (NM_001087442), which was analyzed as a control by using forward primer 5′- AAGAAGGATCTGGCAGCGG-3′ and reverse primer 5′- TTTAATGACACCAGTTTCCACA-3′.

### Statistical analysis

All statistical analyses were carried out using a two-tailed Student’s *t*-test.
